# Focus on post-exertional malaise when approaching ME/CFS in specialist healthcare improves satisfaction and reduces deterioration

**DOI:** 10.3389/fneur.2023.1247698

**Published:** 2023-12-01

**Authors:** Marjon E. A. Wormgoor, Sanne C. Rodenburg

**Affiliations:** ^1^Vestfold Hospital Trust, Division of Mental Health and Addiction, Tønsberg, Norway; ^2^Neuroscience and Cognition, Graduate School of Life Sciences, Faculty of Medicine, Utrecht University, Utrecht, Netherlands

**Keywords:** ME/CFS, myalgic encephalomyelitis, chronic fatigue syndrome, post-exertional malaise, post-exertional symptom exacerbation, patient experience, specialist healthcare, healthcare quality

## Abstract

**Background:**

Post-exertional malaise (PEM) is considered a hallmark characteristic of myalgic encephalomyelitis/chronic fatigue syndrome (ME/CFS). This may also apply to subgroups of patients with long COVID-induced ME/CFS. However, it is uncertain to what extent PEM is acknowledged in routine specialist healthcare for ME/CFS patients, and how this affects patient outcomes.

**Objective:**

This study aims to evaluate to what extent ME/CFS patients experienced focus on PEM in specialist healthcare practice and its significance for outcome and care quality.

**Methods:**

Data from two online cross-sectional surveys covering specialist healthcare services for ME/CFS patients at rehabilitation institutes in Norway and two regional hospitals, respectively, were analyzed. Evaluations of 788 rehabilitation stays, 86 hospital consultations, and 89 hospital interventions were included. Logistic regression models and Mann–Whitney U-tests were used to quantify the impact of addressing PEM on health and functioning, care satisfaction, or benefit. Spearman’s rank correlation and Cronbach’s alpha of focus on PEM with the respondents’ perception of healthcare providers’ knowledge, symptom acknowledgment, and suitability of intervention were assessed as measures for care quality and their internal consistency, respectively.

**Results:**

PEM was addressed in 48% of the rehabilitation stays, 43% of the consultations, and 65% of the hospital interventions. Failure to address PEM roughly doubled the risk of health deterioration, following rehabilitation (OR = 0.39, 95% CI 0.29–0.52; 40.1% vs. 63.2% *P* = <0.001) and hospital intervention (OR = 0.34, 95% CI 0.13–0.89; 22.4% vs. 45.2%, *p* = 0.026). The focus on PEM (PEM-focus) during the clinical contact was associated with significantly higher scores on patients’ rated care satisfaction and benefit of both consultation and intervention. Furthermore, addressing PEM was (inter)related to positive views about healthcare providers’ level of knowledge of ME/CFS, their acknowledgment of symptoms, obtained knowledge, and the perceived suitability of intervention (Cronbach’s alpha ≥0.80).

**Discussion:**

PEM is still frequently not acknowledged in specialist healthcare practice for ME/CFS patients in Norway. Not addressing PEM substantially increased the probability of a decline in health and functioning following the intervention and was strongly associated with reduced perceived care quality, satisfaction, and benefit. These findings may be related to the applied explanatory models for ME/CFS and are most likely of relevance to long COVID.

## 1 Introduction

Myalgic encephalomyelitis/chronic fatigue syndrome (ME/CFS) is a long-term, severe multisystem disease with a distinctive clinical picture, often, but not necessarily, preceded by an infection. Its pathophysiology is still uncertain; therefore, some clinical and research settings apply a biopsychosocial explanatory model for ME/CFS. In these settings, ME/CFS is perceived as a fatigue illness, explained with a psychosomatic understanding as a maladaptive response to an infection or overload, perpetuated by dysfunctional personality factors or beliefs, health anxiety, and deconditioning (1–3). This approach has been criticized for overlooking the evidence of detectable pathophysiological disturbances explaining the symptoms of ME/CFS patients (4–7). Others, however, apply a biomedical approach and consider ME/CFS as a maladaptive pathophysiological response, following an infection or other trigger that remains inadequately studied.

This biomedical explanatory model is acknowledged in the diagnostic criteria sets for ME/CFS that have been defined during the last two decades (6, 8–11). These criteria are more specific than earlier criteria. Core symptoms are fatigue, exertion-induced worsening of disease and symptoms, cognitive dysfunction, and sleep dysfunction (11). Furthermore, immune dysfunction, orthostatic intolerance, neuroendocrine, circulatory, and gastrointestinal dysfunction are common symptoms, while mental illness as the cause of the symptoms is explicitly excluded.

Exertion-induced aggravation of symptoms in ME/CFS is generally called post-exertional malaise (PEM) or post-exertional symptom exacerbation (PESE). It involves a relatively long-lasting and severe worsening of symptoms and/or the appearance of new symptoms, with a further substantial reduction in functioning (9, 12, 13). It may be an immediate or a delayed, disproportionate response to physical, orthostatic, or cognitive effort, or sensory stimuli, which previously were tolerated. It can take days, weeks, or longer to return to baseline (12, 14, 15). Sometimes, a new, more severe baseline is established. The delayed onset and the broad constellation of symptom deteriorations distinguish ME/CFS from other diseases with severe fatigue or deconditioning (6, 16–21). PEM is widely recognized as the most debilitating and persistent feature of ME/CFS (22). The PEM phenomenon has been demonstrated in multiple studies, both with patient-reported outcome measures and with objective measures. Objective findings include new or increased structural and functional abnormalities, following controlled exertion situations (23–26). These findings indicate disturbances in energy metabolism and a dysfunctional autonomic nervous system, impairing the body’s ability to recover from exertion (27, 28). In current practice in Norway, PEM is at best evaluated by anecdotal described experiences; standardized questionnaires (18) or clinical objective PEM, e.g., repeated hand grip strength (29) or cardiopulmonary exercise tests (25), is not routine practice.

If a psychosomatic understanding is applied to approach ME/CFS, PEM is usually disregarded and rather considered as a dysfunctional cognition and extreme behavioral response (30). Interventions typically aim at interrupting the self-perpetuating vicious circle that is thought to maintain symptoms. Assumed mistaken illness beliefs, dysfunctional cognitions, and fear of activity are aimed to be corrected by increasing physical activity to overcome avoidance behavior and regain physical fitness (31, 32). In this view, commonly applied approaches are cognitive behavioral therapy (CBT) and graded exercise therapy (GET), respectively, or varieties that share central conceptual elements.

Current research and clinical recommendations that acknowledge a biomedical base and the PEM phenomenon, however, recognize that there is currently no scientific evidence for effective treatment of ME/CFS and explicitly discourage curative CBT and GET forms (10, 33–37). Instead, pacing strategies are considered to be the most effective approach to reduce the risk of PEM relapse and retain or improve physical functioning and quality of life (10, 36–40).

In Norway, the main responsibility of the diagnosing process of adults with ME/CFS symptoms is held by the general practitioner (GP), preferably a specialist in general medicine (41). In case of unclear differential diagnostic issues, the GP should refer to relevant clinical specialists for further evaluation. The European Network on ME/CFS (EUROMENE) expert consensus (36) recommends referral to specialist service for confirmation of diagnosis, drug treatment, and a range of service offerings, such as multidisciplinary rehabilitation, supportive counseling, education on self-management, and symptom-contingent pacing.

In several studies (42–45), various aspects of perceived care quality in specialist healthcare for ME/CFS patients have been evaluated, but they did not focus on the attention to PEM. In general, only a minority was satisfied with the obtained care and specialists’ knowledge about ME/CFS. In another, recent Norwegian study, however, perceived care quality was evaluated related to the specificity of the diagnosis and PEM severity (42). Patients meeting more specific criteria and patients with higher PEM scores reported more negative experiences with specialist care.

It appears essential to acknowledge PEM in the diagnostic process and therapeutic approach of ME/CFS. To our knowledge, it is inadequately documented to what degree PEM generally is addressed in ordinary healthcare practice or more specifically in specialist healthcare practice in Norway. Likewise, it is insufficiently documented what the consequences are of not addressing PEM for the patient-related outcome and the perceived quality of these services regarding clinical effectiveness, patient safety, and patient experiences.

Awareness and knowledge about PEM seem also of specific relevance for a new growing subgroup of patients facing similar symptoms and biological abnormalities, and PEM (46–52). In the patients with persistent, debilitating symptoms following acute COVID-19 (long COVID), approximately up to half of the patients will meet the diagnostic criteria for ME/CFS (46, 51, 53). Consequently, ME/CFS prevalence is increasing dramatically. Addressing PEM in the approach of long COVID patients is of specific importance as well (54–56).

The aim of this present study was to assess the significance of acknowledging the PEM phenomenon in the clinical approach of ME/CFS patients in specialist healthcare practice.

The first objective was to evaluate to what extent ME/CFS patients experienced focus on PEM during clinical consultations, hospital intervention, or rehabilitation.

The second objective was to estimate to what degree focus on PEM in the received care is related to patient-reported outcomes. The primary outcome is the impact of addressing PEM during an intervention on subsequent changes in health status. The secondary outcome measures are the reported care satisfaction or the perceived general benefit of the obtained care.

The third objective was to assess whether the acknowledgment of PEM in a clinical situation is associated with patient-reported experiences of perceived healthcare quality.

## 2 Materials and methods

This study is a non-prespecified secondary analysis, applying data from two patient surveys executed by the Norwegian ME Association (NMEF). The two patient surveys focused on different healthcare settings, but objectives, methods, and questionnaires are partly similar and described below.

Both surveys were retrospective, anonymous, Internet-based on the platform SurveyMonkey and limited to one response per IP address. There were no time restrictions on response during the study period as the questionnaire remained open until submitted.

The objective of the hospital survey (57) was to evaluate the experiences of ME/CFS patients with specialist healthcare services at two regional hospitals in Southeast Norway. These healthcare services covered two different types of clinical settings: consultations and interventions. Experiences with these settings were evaluated separately in the analyzes. Data collection was performed in the period 5–31 March 2022 and aimed at covering the period since 2017. If they in that period had received care at different departments, the respondents reported that separately, but for each department, only once per consultation and once per intervention.

The rehabilitation survey (58) aimed at retrospectively mapping the experiences of ME/CFS patients with Norwegian rehabilitation services. Data collection was carried out from 4 September 2017 until 15 October 2017 with no restrictions on region and date of stay at one of the rehabilitation facilities.

**Figure 1:**
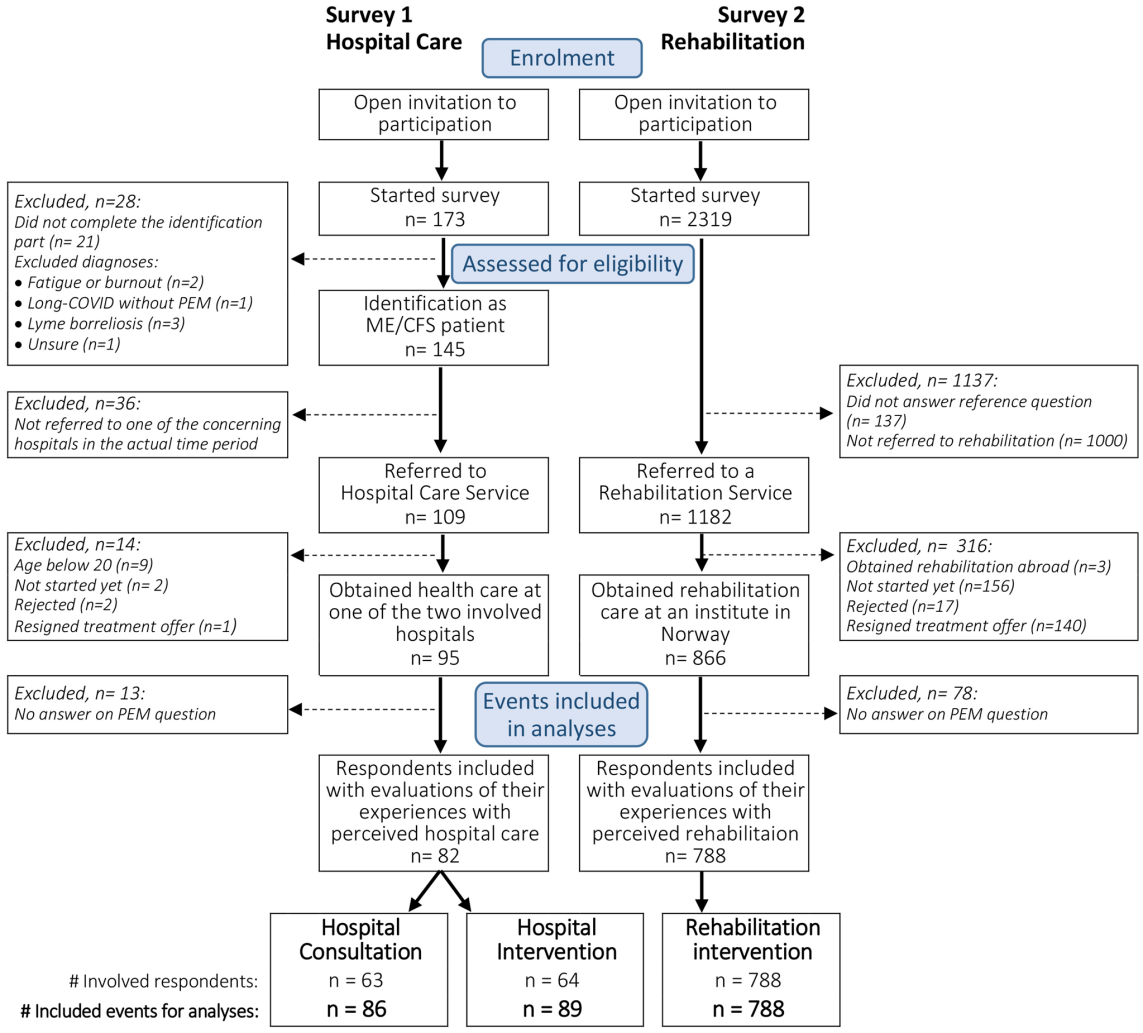
Study flow chart of survey 1 and survey 2, showing the inclusion of respondents and identification of corresponding evaluated care events for analyzes.

For the current study, analyzes were restricted to adult respondents included in the surveys. Respondents should have obtained hospital care at one of the two concerning hospitals or rehabilitation at an institute in Norway. Furthermore, they should have an ME/CFS diagnosis or long COVID with PEM and have answered the question concerning PEM-focus in the obtained healthcare setting. Figure 1 presents the flow chart of the study.

### 2.1 Subjects

For both surveys, invitations were shared on various relevant open and closed Norwegian Facebook groups for ME/CFS patients, their relatives, and other interested parties, both within and outside the ME Association’s auspices. Relatives could answer on behalf of patients who were too ill to answer themselves.

In the hospital survey, members of Vestfold and Telemark Regional ME Association were also directly approached by email. The survey was open for respondents who had been referred to the relevant hospitals during the last 5 years and had an ME/CFS or long COVID diagnosis, were in a diagnosing process for this, or considered themselves as having ME/CFS, post-viral syndrome, or long COVID with PEM. Before evaluating the occurrence of PEM, as well as other typical ME/CFS symptoms, PEM was explained in the survey. Then, the respondents reported which diagnosis they regarded as the most appropriate for them. Only the respondents that answered, “ME/CFS or ME,” “sequela after COVID-19 infection with PEM” or “Post-viral syndrome” and had PEM could progress further in the survey. Respondents not being adults (here below 20) were excluded from the analyzes in the analysis.

In the rehabilitation survey, respondents residing in Norway who previously had obtained an ME/CFS diagnosis G93.3 (59) from the specialist health service or A04 (60) from a GP specialist in general medicine were invited to participate.

### 2.2 Measures

All measures are presented in Tables 1, 2, including applied methods for dichotomization of variables, if relevant. The complete questionnaires (in Norwegian) can be found in the underlying reports (57, 58).

**Table 1:** Relevant questions describing the respondents’ characteristics and the situational context.

Domain	Survey 1 Hospital consultation	Survey 1 Hospital intervention	Survey 2 Rehabilitation
Respondents’ characteristics			
Gender	*Female/male*	*1. Female, 2. male*	-
Age	*< 10, 10–19, 20–29, 30–39, 40–49, 50–59, 60–69, ≥ 70*	*< 10, 10–19, 20–29, 30–39, 40–49, 50–59, 60–69, ≥ 70*	… over 18
Participation work/education	*1. 0%, 2. 25%, 3. 50%, 4. 75%, 5. 100%* *Sickness benefits (≥75%)/ No education lessons*	*1. 0%, 2. 25%, 3. 50%, 4. 75%, 5. 100%* *Sickness benefits (≥75%)/ No education lessons*	
Diagnostician	Who made the diagnosis?	Who made the diagnosis?	Who made the diagnosis?
Disease duration	For how long have you had CFS/ME fatigue symptoms? *< 6 months, 6–12 months, 1–2 years, 2–5 years, 5–10 years, > 10 years*	For how long have you had CFS/ME fatigue symptoms? *< 6 months, 6–12 months, 1–2 years, 2–5 years, 5–10 years, > 10 years*	When was the ME/CFS diagnosis set? (*year*)*—was recalculated to the categories* < 1 year, 1–2 years, 2–5 years, 5–10 years, > 10 years
Disease severity	What severity degree of ME/CFS do you have? 1. *Below mild, 2. Mild, 3, mild–moderate, 4. Moderate, 5. Moderate–severe, 6. Severe, 7. Sever-very severe, 8. Very severe*	What severity degree of ME/CFS do you have? What severity degree of ME/CFS did you have at start of the intervention? *1. Below mild, 2. Mild, 3, mild–moderate, 4. Moderate, 5. Moderate–severe, 6. Severe, 7. Sever-very severe, 8. Very severe*	What was the severity degree at start of the rehabilitation stay? *2. Mild, 4. Moderate, 6. Severe, 8. Very severe*
Situational context			
Medical specialty	Which department or clinic?	Which department or clinic? What sort of intervention?	Which rehabilitation facility?
Intervention duration		How many days? (*open answer*)	How many weeks? *1 /2/3/4/other*
Type of treatment		Counseling, group course, training to increase flexibility, training to increase activity and fitness, relaxation, CBT aimed at coping of severe illness, CBT aimed at symptom reduction and activity increase, medications or supplements, or other	
Group course-target patient group		ME/CFS, fatigue, or various health complaints	

Answer alternatives are italicized.

**Table 2:** Relevant questions that were applied in the analyzes: PEM-focus in the clinical contacts, variables assessing patient-reported outcome, and patient-reported experiences of perceived healthcare quality.

Domain	Survey 1 Hospital consultation	Survey 1 Hospital intervention	Survey 2 Rehabilitation
Post-exertional malaise (PEM)			
PEM-focus	Were you asked, directly or indirectly, if you had PEM? *1. No, 3. Unsure, 5. Yes*	Did you gain any new knowledge or understanding about PEM?	Was PEM explained during the stay? *1. No, 5. Yes*
*1. PEM was not seen as typical or relevant, 2. No information, 3. Nothing new, 4. Some, 5. A lot*			
Patient-reported outcome			
Care satisfaction/Benefit	Overall, were you satisfied with the consultation? **	What benefit have you had, overall, from the intervention? *1. No benefit, 2. Little benefit, 3. Some benefit, 4. Large benefit, 5. Very large benefit*	I am satisfied with my stay at the rehabilitation facility *
Impact on health		What severity degree of ME/CFS did you have the first following 2 weeks/the following 3 to 6 months? *1. below mild, 2. Mild, 3. Mild–moderate, 4. Moderate, 5. Moderate–severe, 6. Severe, 7. Sever-very severe, 8. Very severe*	I felt better just after the stay than before I felt better 1 month after the stay than before *1. Strongly disagree, 2. Disagree, 3. Neither agree nor disagree, 4. Agree, 5. Strongly agree*
Impact on various domains		How did you benefit from the intervention, when it comes to: physical health, cognitive effort, mental health, and ability to master daily tasks, ability to regulate activity level, quality of life? *1. Much worse, 2. Somewhat worse, 3. No, change, 4. Somewhat improved, 5. Strongly improved*	
Patient-reported experiences of perceived healthcare quality			
Suitability of the intervention		Did you feel that the intervention was suitable for your situation? *	The activity level was adapted to my illness*
Healthcare provider knowledge*^?^*	Do you think that the doctor or possibly other healthcare provider had a good knowledge of M//CFS? *1. Very little, 2. Not much, 3. Both, 4. Good, 5. Very good*	Do you think that this therapist/supervisor/institution had good knowledge of ME/CFS? 1. Very *little, 2. Not much, 3. Both, 4. Good, 5. Very good*	The healthcare providers had good knowledge on ME/CFS*
Symptom acknowledgment*^?^*	Did you feel that your symptoms were taken seriously? *	Did you feel that your symptoms were taken seriously? **	The staff at the rehabilitation facility were understanding when I told them about my symptoms *
Gained beneficial knowledge or skills		Did the intervention help you to be able to prevent and manage PEM? 1. *PEM was not seen as typical or relevant, 2. No information, 3. Nothing, 4. Somewhat, 5. A lot*	I learned a lot that I have benefited from later *
Incorrect treatment		Do you think you obtained incorrect treatment in some way?**	–
Intervention completed		Did you complete the intervention? 1. *No I quit because I got worse/ no I quit because I did not think it was helpful, 2. Yes*	Were you at the rehabilitation facility for the entire period? *1. No, I got worse and chose to go home/ no, I got worse and was sent home by the staff, 2. Yes*

Answer alternatives are italicized, and in the case of dichotomized response options, the desirable responses are underlined. *1. Strongly disagree, 2. Disagree, 3. Neither agree nor disagree, 4. Agree, 5. Strongly agree; **1. Not at all, 2. To a small degree, 3. To some degree, 4. To a great degree, 5. To a very great degree.

#### 2.2.1 Respondent characteristics

The operationalization of respondents’ characteristics is presented in Table 1. Sociodemographic characteristics covering gender, age, and participation degree in work and school were only evaluated in the hospital survey. Both surveys evaluated some disease characteristics such as diagnosis, disease duration, and severity grading.

#### 2.2.2 Post-exertional malaise-focus as an explanatory variable

The primary variable of interest was the focus on PEM (PEM-focus) in specialist healthcare settings and its impact. PEM-focus in the three types of healthcare settings was operationalized with closed questions, but with different wording and different scales for each type of healthcare setting (see Table 2). In the analyzes, PEM-focus was dichotomized as PEM+ (PEM was addressed) or no-PEM (PEM was not addressed); this is described in Table 2 as well.

#### 2.2.3 Patient-reported outcome

The assessments of the outcome measures are presented in Table 2. The impact of hospital intervention on health status was operationalized by computing changes in the evaluated disease severity before and after the intervention–in the first 2 weeks (post-intervention) and 3 to 6 months (short-term follow-up). Disease severity classification was based on the Norwegian National Guidelines for CFS/ME (41) and ICC (9). More severe disease severity after the intervention was classified as ‘deteriorated’. ‘Not deteriorated’ includes both unchanged and improved health status. In addition, the benefit of the hospital interventions was more specifically evaluated related to various domains: physical health, cognitive effort, mental health, ability to master daily tasks, ability to regulate activity level, and quality of life. The answer options “much worse” and “somewhat worse” were rated as “deteriorated.”

Post-intervention changes in health status following rehabilitation were operationalized as ‘deteriorated’ if the respondents strongly disagreed with the statement “*I felt healthier just after the stay than before*.” Short-term changes reported as “*I felt better one month after the stay than before*” were considered as “deteriorated” if the respondent strongly disagreed. Other replies were valued as ‘not deteriorated’.

In addition, satisfaction with the consultation or the rehabilitation program and perceived general benefit of the hospital intervention were assessed with 5-point Likert scales and applied as outcome measures.

#### 2.2.4 Patient-reported experiences of perceived healthcare quality

Relevant items are presented in Table 2. In addition to treatment completion, all care quality variables were assessed with 5-point Likert scales. Operationalization varies by care setting. Appraisal of the quality of the clinical consultations (the hospital survey) was assessed by evaluating the patients’ view on the ME/CFS-specific knowledge and experienced symptom with respect to the healthcare professionals. Rated suitability of the intervention to the respondents’ condition and the proportion who completed treatment were considered as additional indicators for care quality of intervention and rehabilitation. An item of the rehabilitation survey that evaluated whether the respondents felt they had obtained useful knowledge was included. For hospital intervention, respondents’ opinion of the extent to which they had acquired PEM coping skills and whether they had obtained incorrect treatment was included as well.

#### 2.2.5 Situational context

In both surveys, the intervention duration and the involved hospital, department, or rehabilitation institution were assessed with closed questions and an ‘other’ option (see Table 1).

In the hospital survey, the type of intervention options was assessed systematically as well: individual treatment, group course, or both. The types of treatment options were exercises to increase mobility, aerobic condition, or relaxation, cognitive behavioral therapy (CBT) aimed at reducing symptom focus and increasing activity, CBT focused on support and illness coping, or medication.

Apart from which particular rehabilitation institution was evaluated, no context variables were assessed systematically in the rehabilitation survey.

### 2.3 Analysis

Analyzes are based on available data from two surveys: a hospital survey (57) and a rehabilitation survey (58).

Perceived PEM-focus (PEM+ or no-PEM) in provided specialist healthcare is the main object of interest. For the different healthcare settings, PEM-focus, as evaluated by the respondents, is mainly analyzed as dichotomized variables.

Situational context variables, such as which hospital, department, or rehabilitation institution, as well as type of intervention, are not presented in detail. General context differences in PEM-focus were evaluated with chi-square tests.

To determine the impact of PEM-focus on the outcome, binary univariate and multivariate logistic regression were used with PEM-focus as the explanatory variable and disease duration and severity (14, 61, 62) included as covariates if available. The response variables were dichotomized outcome measures of satisfaction or rated general benefit, impact on health status following the intervention, and additionally for the hospital interventions, the impact on various ME/CFS-related domains. Because of the limited expected improvement in health status and the real possibility of deterioration following the intervention, health impact was evaluated as ‘no-deterioration’ versus ‘deterioration’.

Satisfaction with clinical consults was only evaluated with univariate analyzes; crude odds ratios (ORs) with corresponding 95% confidence intervals (95% CI) are presented. For both intervention settings, both crude and adjusted OR were calculated. Hence, OR > 1.0 indicates that the variable is associated with a higher probability of the response variable (satisfaction, benefit, health, or function deterioration), whereas OR < 1.0 indicates an association with a lower probability. The results were also presented as bar diagrams with full-scale outcome variables. Mann–Whitney U-test was applied to assess group differences. The impact of PEM-focus on changes in health status following hospital intervention was evaluated with paired-sample Wilcoxon signed rank-sum tests for PEM+ and no-PEM.

The care quality variables are presented with Spearman’s rank correlation coefficient (Spearman’s rho: ρ) as a measure of association with PEM-focus. In addition, Cronbach’s alpha was calculated as a measure of internal consistency of the care quality variables and the full scale of PEM-focus answers.

No power calculation was performed as the primary surveys were considered explorative. Differences between respondents that were included and excluded in the analyzes of this study are compared in the available disease characteristics within both surveys with chi-square tests. In the hospital survey, screening for ME/CFS diagnosis and possible exclusion if ME/CFS was not considered as their main diagnosis was done in the first part of the survey. Sociodemographics were questioned at the end of the survey and thus were not answered by most of the excluded respondents. In the rehabilitation survey, no sociodemographic characteristics were collected. This made it impossible to compare the sociodemographics of respondents who completed vs. not completed the surveys.

Data analyzes were performed using IBM SPSS Statistics for Windows, version 28 (IBM Corp., Armonk, N.Y., United States). A *p-*value of less than 0.05 was considered statistically significant.

## 3 Results

### 3.1 Subjects

Figure 1 shows the study flow chart including both surveys. In the hospital survey, 82 respondents were included in the analyzes. In total, 86 consultations and 89 interventions were evaluated. The majority had evaluated only one consultation (71%) or intervention (69%), 21 and 23%, respectively, had evaluated two, and 8% had evaluated consultations and interventions with three different departments. In the rehabilitation survey, 788 respondents who had participated in a rehabilitation program at a rehabilitation facility in Norway were included.

The non-completers of the hospital survey did not differ in illness duration, age of symptom debut, diagnosis and disease severity, the degree they experienced PEM, and fulfillment of the Canadian Consensus Criteria (8) as evaluated in this survey. The non-completers had less often obtained an ME/CFS or long COVID with PEM diagnosis (80.5% vs. 97.5%, *p* = 0.002). In the rehabilitation survey, there was no difference in how long ago the diagnosis was set and by whom, between the respondents that were included or excluded for analyzes (see Figure 1).

#### 3.1.1 Sociodemographic and disease characteristics

In the hospital survey, 84.5% was female and the age distribution at the time of the survey was 26.4% 20–29 yr., 18.1% 30–39 yr., 27.8% 40–49 yr., 26.4% 50–59 yr., and one respondent older than 60. The majority (88.7%) was not working or studying at all, 5.6% worked or studied 1–5 h weekly, 4.2% 6–20 h, and only one respondent worked or studied more than 20 h weekly. There were no sociodemographic data available from the respondents of the rehabilitation survey.

ME/CFS was self-reported as the main diagnosis by 80 of the 82 respondents of the hospital survey; for 79, a physician had set this diagnosis as well. One respondent had obtained a ‘burnout or chronic fatigue’ diagnosis. Two respondents had long COVID; this was confirmed for one respondent. In total, 24% of the respondents were diagnosed by a GP only, 39% by a specialist of one of the hospitals only, or by other specialists in private practice only (7%). The remaining respondents were diagnosed by both a GP and a specialist (24%) or by both a hospital and a private specialist (5%). No respondents had reported that they ‘had a fatigue illness (including ME/CFS) before but not now’.

All patients in the rehabilitation survey self-reported that they had been diagnosed with ME/CFS. Twenty patients (0.9%) of the subjects that had started the survey reported they were neither a ME/CFS patient nor a relative and had been excluded. 20% of the respondents had received the diagnosis from their GP, 23% had consulted a specialist in private practice, and 49% had received the diagnosis from the local or regional hospital. 8% had received the diagnosis from the national CFS/ME center, a third-line service for advanced interdisciplinary assessment and guidance for adult patients.

Disease duration and severity are reported in Table 3.

**Table 3:** Disease duration and severity.

	Hospital survey		Rehabilitation survey	
	*n*	(%)	*n*	(%)
ME disease duration*	82		770	
< 1 yr.	0	0.0%	21	2.7%
1–2 yr.	5	6.1%	182	23.7%
2–5 yr.	19	23.2%	247	32.2%
5–10 yr.	33	40.2%	217	28.3%
> 10 yr.	25	30.5%	100	13.0%
Disease severity at intervention start	52		788	
Mild	2	3.7%	162	20.6%
Mild to moderate	20	37.0%		
Moderate	17	31.5%	519	65.9%
Moderate to severe	12	22.2%		
Severe	3	5.6%	106	13.5%
Severe to very severe	0	0.0%		
Very Severe	0	0.0%	1	0.1%

*Hospital survey: duration of ME/CFS symptoms at the date of survey response. Rehabilitation survey: time from year of diagnosis until intervention start.

### 3.2 Situational context and PEM-focus

The clinical consultations with a health provider at the two relevant hospitals (*n* = 86) were received at mainly six different types of departments. The majority had been at an ME/CFS Medicine Clinic (30.2%), a department of Physical Medicine (23.3%), or Neurology (20.9%). The others had been at a department of Infectious Diseases (8.1%), Mental Health (8.1%), or an ME/CFS Outpatient Clinic (5.8%), Gastroenterology (2.3%), or Pulmonary (1.2%). The hospital interventions (*n* = 89) were mainly received at a department of Physical Medicine (38.2%) or a department for Therapeutic Patient Education (38.2%). The remaining interventions were received at an ME/CFS Medicine Clinic (6.7%), departments of Mental Health (6.7%), Health and Work (5.6%), or Neurology (4.5%).

The type and duration of the hospital interventions varied. Intervention could include educational group courses (70.8%), individual consultation/one-to-one counseling (57.3%), or both. In addition to education in the group courses, the interventions comprised CBT aimed at reducing symptom focus and increasing activity (14.6%), CBT focused on support and illness coping (11.2%), exercises to increase mobility (4.5%), aerobic condition (3.4%), or relaxation (4.5%), as well as medication or dietary supplements (2.2%). Most hospital interventions were delivered on an outpatient basis, generally once or a few times. The educational courses were either intensive (3 days within 1 week) or spread over a longer period (6–8 times, once every 1 or 2 weeks). Only 67.2% of the respondents attended educational courses aimed specifically at ME/CFS, and the rest of the courses were aimed at patients with either general fatigue (21.8%) or other health complaints (10.9%).

Experiences of obtained rehabilitation services (*n* = 788) were evaluated for over 20 rehabilitation facilities in Norway. Two rehabilitation facilities were each evaluated by over 100 respondents (32.1% of respondents), four by over 50 respondents (35.3%), and four by at least 20 respondents. In the rehabilitation survey, applied intervention methods were not evaluated systematically. However, according to the open-ended comments in the survey, the rehabilitation institutions had different approaches to the rehabilitation of ME/CFS patients. Some encouraged CBT aimed at reducing symptom focus combined with a graded activity increase. Other rehabilitation facilities provided explanations about exertion-induced symptom exacerbation (PEM) and focused on the importance of managing and adjusting activity levels according to the patient’s capacity (“energy envelope theory”) to prevent PEM (58).

**Figure 2:**
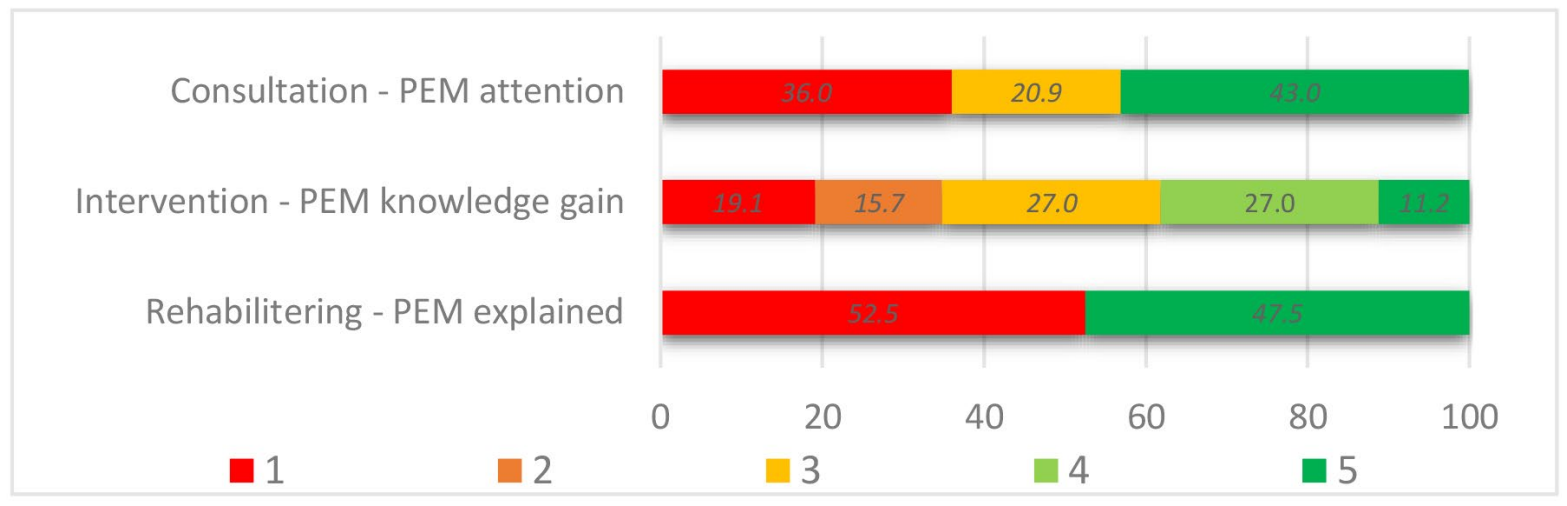
PEM-focus in the healthcare settings. Answer options of Assessment of PEM, in consultation (*n* = 86): 1. No (no-PEM), 3. Unsure (no-PEM), 5. Yes (PEM+). Knowledge gain following hospital intervention (*n* = 89): 1. PEM was not seen as typical or relevant (no-PEM), 2. No information (no-PEM) n, 3. Yes, but nothing new (PEM+), 4. Some (PEM+), 5. A lot (PEM+). PEM explained in rehabilitation setting (*n* = 788): 1. No (no-PEM), 5. Yes (PEM+).

Overall, respondents reported that PEM was addressed in 43.0% of the consultations, 65.2% of the hospital interventions, and 47.5% of the rehabilitation stays. A more detailed distribution is presented in Figure 2. Whether PEM was addressed (PEM+) or not (no-PEM) varied significantly across the different settings from zero to 81% for clinical consultations (*p* < 0.001) and from 29 to 100% for hospital intervention (*p* < 0.001). Among the respondents who had participated in an educational group course or had received counseling, 71.4 and 45.1%, respectively, reported that PEM had been addressed. In the group courses specifically aimed at ME/CFS patients, 97.7% perceived PEM+, while PEM+ was 14.3% in the groups for fatigue and other health complaints. Among the rehabilitation facilities, reported PEM+ varied significantly as well, from 2.2 to 68.8% (*p* < 0.001).

### 3.3 Post-exertional malaise-focus as an explanatory variable for the outcome

**Figure 3:**
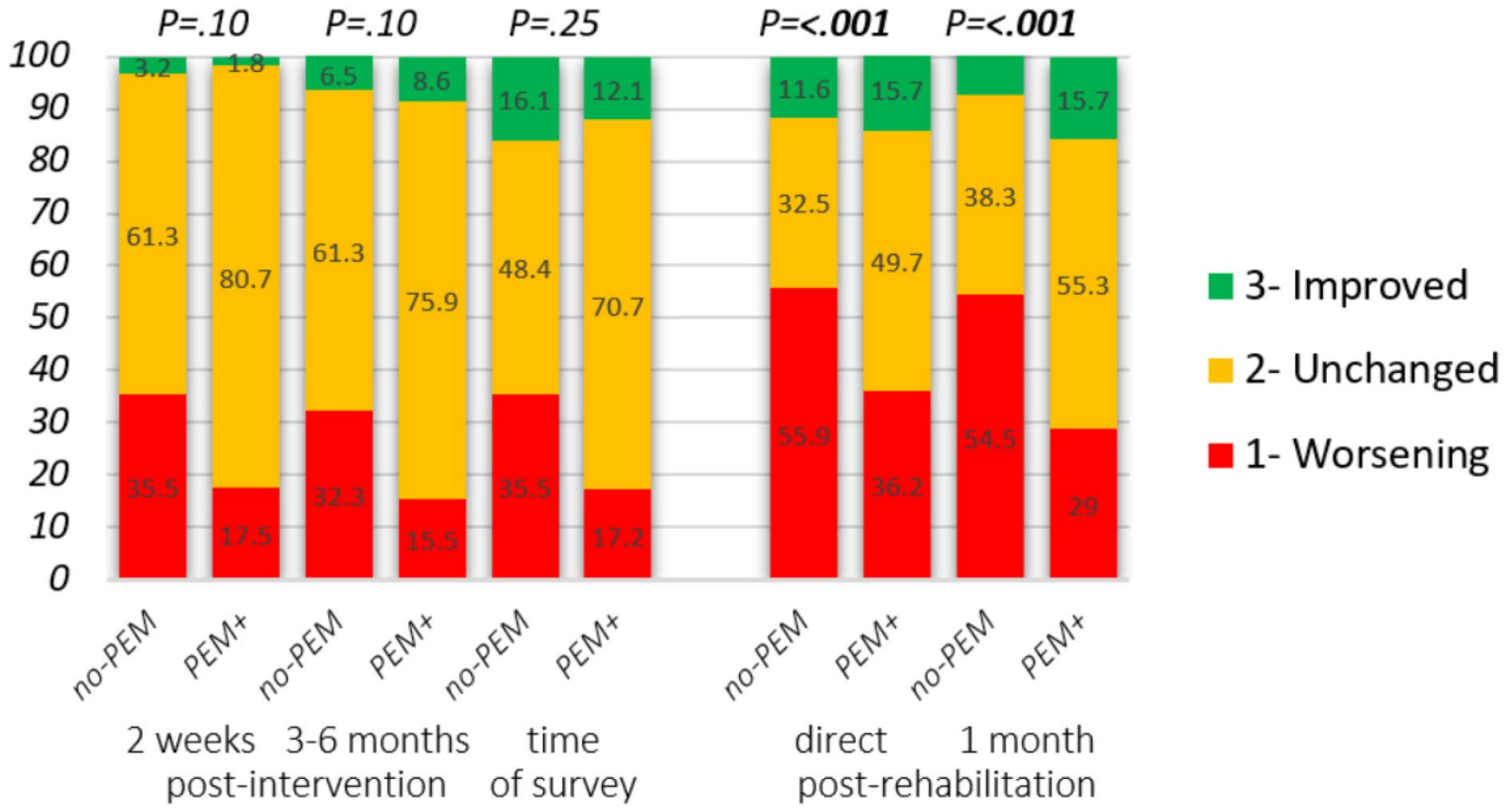
Impact of hospital intervention and rehabilitation on the state of health stratified by PEM-focus in the therapeutic approach. Changes from intervention start. Hospital intervention: self-reported severity degree at baseline, 2 weeks (*n* = 88), and 3 to 6 months (*n* = 89) following the intervention. Change in clinical severity degree: 1. Higher disease degree, 2. Unchanged, 3. Lower disease degree. Rehabilitation: reply to the statements “I felt better just after the stay than before” and “I felt better 1 month after the stay than before,” answer options: 1. Strongly disagree, 2. Disagree/neither agree nor disagree, 3. Strongly agree. Mann–Whitney U-test was applied to assess group differences.

**Figure 4:**
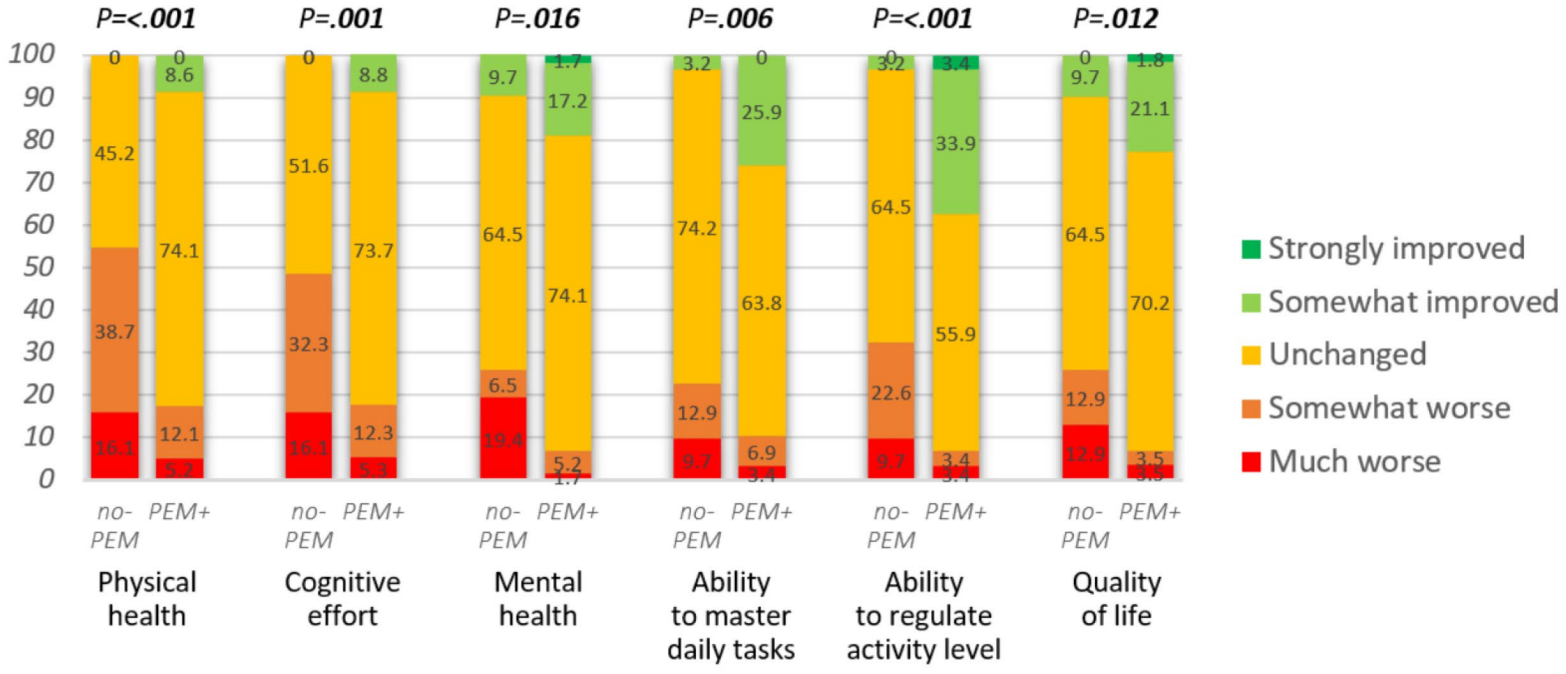
Impact of hospital intervention on various domains (*n* = 88 or 89). Mann–Whitney U-test was applied to assess the group differences.

**Figure 5:**
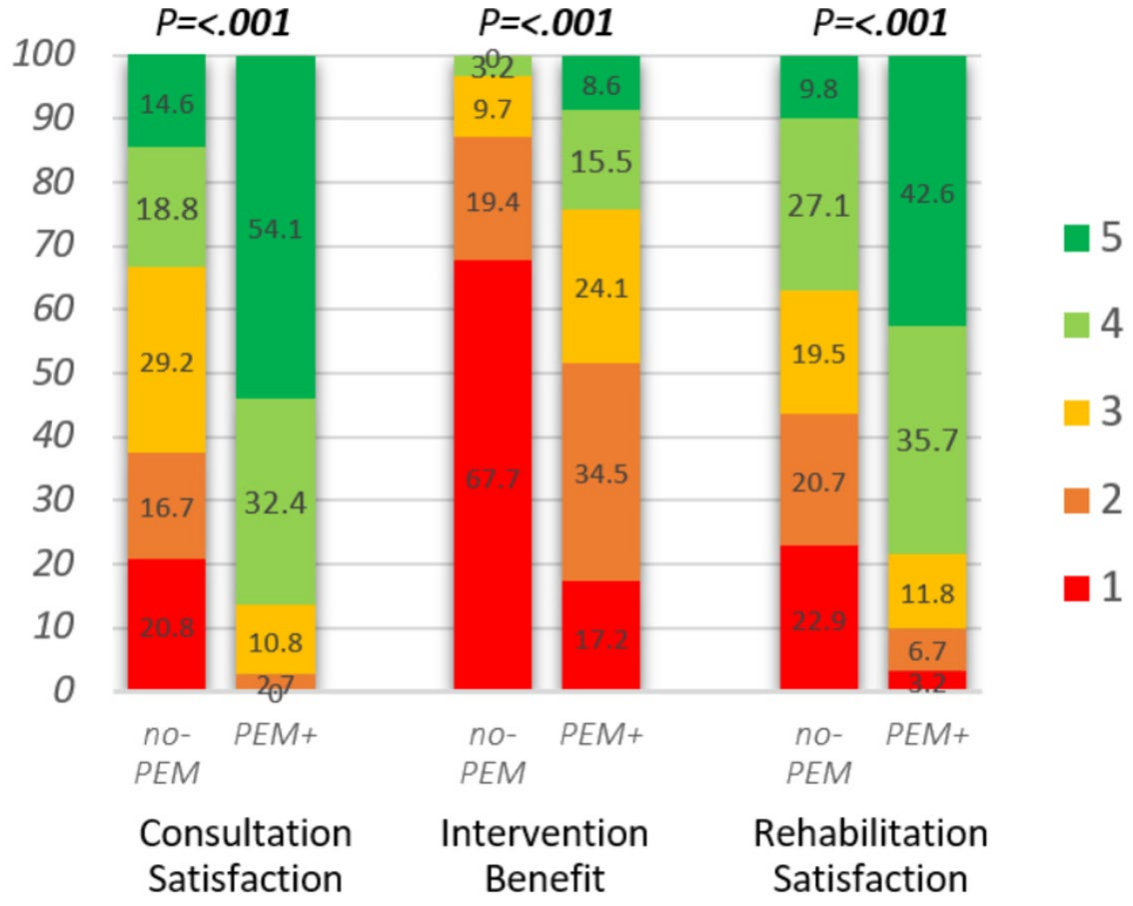
Impact of hospital consultation (*n* = 85), intervention (*n* = 89), and rehabilitation (*n* = 783) on rated satisfaction or benefit, stratified by PEM-focus during clinical contact. Consultation satisfaction: “All in all, were you satisfied with the consultation?,” answer options: 1. not at all, 2. to a small degree, 3. to some degree, 4. to a great degree, 5. to a very great degree. Intervention benefit, hospital intervention: “What benefit have you had, all in all, from the intervention?” answer options: 1. No, 2. Little, 3. Some, 4. Large, 5. Very large. Rehabilitation satisfaction: “I am satisfied with my stay at the rehabilitation facility,” answer options: 1. Strongly disagree, 2. Disagree, 3. Neither agree nor disagree, 4. Agree, 5. Strongly agree. Mann–Whitney U-test was applied to assess the differences between no-PEM and PEM+.

Differences in several outcome measures stratified by PEM-focus (no-PEM or PEM+) are presented in Figures 3–5. In addition, Table 4 summarizes the results of logistic regression analyzes for the association between PEM-focus and binary outcome measures. Multivariate logistic regression analyzes produced nearly identical results as univariate logistic regression for the impact of PEM. The results of the univariate regression analyzes are, therefore, not presented here.

**Table 4:** Results of logistic regression analysis for the association between PEM-focus (no-PEM or PEM+) and outcome.

Setting	*n*	Response variables (Outcome)	Explanatory variables	OR	[95% CI]	*p*
Hospital consultation	79	Satisfaction	PEM-focus	11.57	[3.72–35.96]	**<0.001**
Hospital intervention	89	Benefit	PEM-focus	9.74	[1,21–78.57]	**0.033**
			Disease severity^a^	0.93	[0.83–1.05]	0.26
	89	Function deterioration^b^	PEM-focus	0.13	[0.05–0.37]	**<0.001**
			Disease severity^a^	1.12	[1.01–1.24]	**0.034**
	88	Worsening disease severity- post, 1–2 wk^c^	PEM-focus	0.37	[0.14–1.04]	0.058
			Disease severity^a^	0.93	[0.84–1.03]	0.166
	89	Worsening disease severity- 3-6 mos^c^	PEM-focus	0.38	[0.14–1.08]	0.07
			Disease severity^a^	0.97	[0.88–1.08]	0.57
Rehabilitation	742	Satisfaction	PEM-focus	5.75	[4.14–7.98]	**<0.001**
			Disease severity^a^	0.63	[0.48–0.84]	**0.002**
			Disease duration	0.99	[0.94–1,05]	0.77
	768	Worsening health–post	PEM-focus	0.46	[0.34–0.63]	**<0.001**
			Disease severity^a^	1.29	[1.00–1.67]	0.052
			Disease duration	0.98	[0.93–1.03]	0.44
	769	Worsening health–1 mo.	PEM-focus	0.35	[0.26–0.48]	**<0.001**
			Disease severity^a^	1.48	[1.13–1,94]	**0.005**
			Disease duration	0.99	[0.94–1.05]	0.83

Relevant disease variables were included as covariates, if available. ‘No-PEM’ is the reference category for PEM focus. ^a^Disease severity at intervention start; ^b^Worsening physical, cognitive, or mental functioning; ^c^changes in disease severity compared to baseline.

Figure 3 and Table 4 present the impact of PEM-focus on the health state after finishing the intervention. For the majority, their health state did not change. On average, for respondents in both groups, disease severity was worsened in the first 2 weeks following hospital intervention (*p* = 0.005 in no-PEM and *p* = 0.008 in PEM+). From baseline until 3 to 6 months following baseline, differences were only significant in no-PEM (*p* = 0.042 and *p* = 0.13 in PEM+).

However, there was a tendency that at both time points, around twice as many respondents from the no-PEM group experienced a deterioration of health status, following the intervention compared to the PEM+ group. Overall, if PEM had not been addressed in the intervention, logistic regression showed that the odds of experiencing health deterioration on at least one of the two time points increased significantly following both hospital intervention (proportion 22.4% in PEM+ vs. 45.2% in no-PEM, *p* = 0.026) and rehabilitation (40.1% vs. 63.2% *P =* <0.001) [adjusted OR: 0.34 (95% CI 0.13–0.89; *p = 0.027*) and 0.39 (95% CI 0.29–0.52; *P* = <0.001), respectively]. At the time of data collection (up to 5 years after hospital intervention), 35.5% of no-PEM respondents and 17.2% of PEM+ respondents had a more severe disease degree (OR 0.38, 95% CI 0.14–1.03, *p* = 0.058) compared to the start of the intervention. In the rehabilitation survey, changes in disease severity were not assessed.

The lack of focus on PEM in the hospital intervention had a significant impact on physical and mental health, cognitive effort, ability to master daily tasks, ability to regulate activity level, and quality of life (see Figure 4; Table 4). The respondents from the no-PEM group experienced over three times more often any physical, cognitive, or mental function worsening following hospital intervention [61.3% vs. 19.0%, *p* < 0.001, adjusted OR = 0.13 (95% CI 0.05–0.37), *p* < 0.001].

Figure 5 and Table 4 show the treatment outcome assessed as patient satisfaction or benefit following hospital consultations, interventions, or rehabilitation, stratified on PEM-focus. Evaluated satisfaction or benefit was generally significantly higher (all *p* < 0.001) in all three clinical settings when PEM was addressed. Satisfaction with consultation and rehabilitation was twice as high and over 4-fold as many respondents reported to have perceived at least some benefit of the hospital intervention.

In the educational group courses at the hospitals, outcome measures were strongly related to the specificity of the intervention. Deterioration of health and functioning and perceived benefit was significantly less frequently reported after the ME/CFS-specific courses, compared to the courses for general fatigue or health complaints. Worsening of health was experienced by 25.6% vs. 52.4% (*p* = 0.034), and deterioration of physical, cognitive, or mental function was reported by 18.6% vs. 71.4% (*p* < 0.001). Perceived benefit was low in both groups: 23.3% of the respondents that had participated in ME/CFS-specific courses and 9.5% of the participants of less specific courses (*p* = 0.19) had reported large or very large benefits of the education.

### 3.4 Care quality related to PEM-focus

Table 5 presents the correlation between focus on PEM in different clinical situations and care quality as perceived by the respondents. In all three types of healthcare settings, respondents’ perceptions of the healthcare provider’s level of ME/CFS knowledge and symptom acknowledgment were strongly associated with whether or not there had been attention to PEM. PEM-focus in hospital intervention and rehabilitation was also strongly correlated with respondents’ opinion on whether the intervention was suitable and sufficiently adjusted to their situation. Cronbach’s alpha of respective 0.89, 0.89, and 0.80 of the care quality variables and PEM-focus indicates high internal consistency.

**Table 5:** Distribution of degree of perceived care quality on several factors as reported by the respondents in the three types of care settings, stratified and tested by PEM-focus. Measures for internal consistencies calculated of all variables, including PEM-focus, are presented as well.

	Survey 1—hospital PEM-focus in consultation				Survey 1–hospital PEM-focus in intervention				Survey 2–rehabilitation PEM-focus in rehabilitation			
	*n*	no-PEM 49 (56%)	PEM+ 38 (44%)	*P*-value	*n*	no-PEM 31 (35%)	PEM+ 58 (65%)	*P*-value	*n*	no-PEM 414 (53%)	PEM+ 374 (48%)	*P*-value
		*Test statistics*				*Test statistics*				*Test statistics*		
Healthcare provider ME/CFS knowledge	86	*ρ* = 0.62		<0.001	89	*ρ* = 0.74		<0.001	786	*ρ* = 0.56		<0.001
Very little	11	22.4%	0.0%		18	51.6%	3.4%		125	28.4%	2.1%	
Not much	10	18.4%	2.7%		9	25.8%	1.7%		115	23.3%	5.1%	
Both	17	28.6%	8.1%		17	19.4%	19.0%		125	19.2%	12.3%	
Good	16	16.3%	21.6%		23	3.2%	37.9%		228	21.1%	37.6%	
Very good	32	14.3%	67.6%		22	0.0%	37.9%		193	8.0%	42.8%	
Symptom acknowledgment	86	*ρ* = 0.60		<0.001	88	*ρ* = 0.55		<0.001	784	*ρ* = 0.48		<0.001
Not at all	10	20.4%	0.0%		12	30.0%	5.2%		73	16.8%	1.1%	
To a small degree	9	18.4%	0.0%		9	23.3%	3.4%		72	14.1%	3.8%	
To some degree	13	22.4%	5.4%		17	23.3%	17.2%		84	16.1%	4.8%	
To a great degree	17	18.4%	21.6%		26	20.0%	34.5%		275	36.0%	34.0%	
To a very great degree	37	20.4%	73.0%		24	3.3%	39.7%		280	17.0%	56.3%	
Suitability of intervention					89	*ρ* = 0.61		<0.001	785	*ρ* = 0.46		<0.001
Not at all					17	45.2%	5.2%		161	34.2%	5.4%	
To a small degree					10	22.6%	5.2%		127	18.9%	13.1%	
To some degree					22	22.6%	25.9%		113	17.7%	10.7%	
To a great degree					24	6.5%	37.9%		243	22.3%	40.5%	
To a very great degree					16	3.2%	25.9%		141	6.8%	30.3%	
Completed intervention					78	*ρ* = 0.26		0.021	784	*ρ* = 0.06		0.12
No					12	27.6%	8.2%		104	15.0%	11.3%	
Yes					66	72.4%	91.8%		680	85.0%	88.7%	
Cronbach’s alpha		0.89				0.89				0.80		

Spearman’s rank correlation coefficient (Spearman’s rho: ρ) as a measure of association with PEM-focus. Cronbach’s alpha as a measure of internal consistency (with the full scale of PEM-focus).

During rehabilitation, 28.4% of the no-PEM respondents vs. 73.5% of the PEM+ respondents (*p* < 0.001) had learned a lot which they had benefited from afterward. In hospital intervention, none of the no-PEM respondents versus 58.6% (*p* < 0.001) of PEM+ had obtained new knowledge or understanding about PEM, and 3.3% of no-PEM vs. 46.5% of PEM+ (*p* < 0.001) had obtained new PEM coping skills. Almost half (48.4%) of the no-PEM group vs. 5.2% (*p* < 0.001) of the PEM+ group felt that they had been treated incorrectly in the intervention obtained at the hospital.

For the educational group courses, most care quality measures were also strongly correlated with whether the target group was specific for ME/CFS patients or not [healthcare providers’ ME/CFS knowledge, *ρ* = 0.64 (*p* < 0.001); symptom acknowledgment, *p*  = 0.59 (*p* < 0.001); and suitability of intervention, *ρ* = 0.63 (*p* < 0.001)]. There were, however, no significant differences in dropout ratios: 5.3% in the ME/CFS-specific education and 10.0% in the other courses (*p* = 0.50).

## 4 Discussion

The PEM phenomenon is a hallmark feature of ME/CFS and essential to acknowledge in both clinical consultation and intervention. This study was conducted in Norway, generally featuring high-quality care. Nevertheless, according to a significant proportion of the ME/CFS patients, PEM had frequently not been addressed during their contact with specialist healthcare services. This concerned both consultation services at the hospitals as well as the interventions delivered at the hospitals and rehabilitation institutions. This lack of focus on PEM increased the probability of experiencing deterioration, following hospital intervention and rehabilitation care. On the other hand, addressing PEM was related to increased rated care satisfaction, healthcare quality, and benefit.

### 4.1 Addressing PEM in the intervention

Over one-third of the respondents of the hospital interventions and half of the individuals who had stayed at a rehabilitation institute reported that PEM had not been addressed. This doubled the number of respondents that acquired a more severe disease degree for a long time; for the hospital respondents, the data demonstrated that these differences were still present at the time of data collection (i.e., up to 5 years after the intervention).

From a psychosomatic point of view (3, 63, 64), PEM is ignored as a direct physiological response to physical or mental exertion. From this perspective, GET and CBT are considered as effective therapies. Although the Norwegian Guidelines regard PEM as a cardinal symptom, GET and CBT are still suggested as effective treatment approaches in these guidelines (41). This is despite there currently being no research evidence of convincing effects of these approaches for ME/CFS patients with PEM (10, 33, 34, 65, 66) and despite the fact that several surveys actually reported that over half of the ME/CFS patients experience substantial deterioration after GET and usually do no benefit from CBT (67–69).

GET and curative CBT were seldom explicitly mentioned as applied method in both our hospital and rehabilitation surveys. Yet, many patients reported that they encountered elements of CBT and GET, such as being encouraged to believe their disease is not serious or physical, encouragement to increase activity levels, and disregarding symptoms. This usually happened in settings where PEM was not addressed. Some of the citations that the respondents had added in comments text fields in both surveys testify to this (see Table 6).

**Table 6:** Illustrating citations of the respondents of both underlying surveys (freely translated from Norwegian) (57, 58).

Psychosomatic approach
*“The doctor said it should not be called ME but rather ‘BE’ because it is Between the Ears.”* *“We were met by a psychologist who claimed that if you felt exhaustion coming over you, you should think of something pleasant and that ‘feeling’ would go away!.”* *“There was a great deal of focus on stress management and stuck-thought- patterns.”* *“I felt that ME was not taken very seriously; all forms of exhaustion seemed to be taken under the same umbrella.”* *“PEM and exhaustion were seen as complaints and depression, and as an excuse not to exercise.”*
Sustained arousal hypothesis
*“The doctor believed that I could recover completely with their approach; a sustained stress response, which is cured with the right mind-set and individually adapted training.”* *“They were only concerned with the body’s stress response.”*
Consequences of opposing explanatory models of ME/CFS
*“Because of their perception that ME comes from a biopsychosocial model of explanation, I was never able to become fully comfortable with them. I have a completely different experience of the disease and they focused far too much on the psychological side.”*
Poor disease understanding among healthcare providers
*“The healthcare providers barely knew anything about ME/CFS, but they tried their best. The stay was too much. Just being there. It took many years for me to get back to the same level I was before I left.”*
Ignoring symptoms
*“I told them about all the symptoms, but was then told that we had too much focus on symptoms.”* *“It was just about not thinking about the symptoms, that you get well as long as you increase your activity and think positively.”* *“The (rehabilitation) stay is based on CBT and GET, the patient himself must be well aware of his own limits, otherwise it can become too much.”*
Addressing PEM perceived as more positively
*“It was a very nice stay and it was nice to meet more people like me. I did not get any better, but I brought home some tips on everyday life that make it a little easier.”* *“The first time I met healthcare providers who believed in me and took my illness into account”* *“If you could not handle an activity, they said, ‘It’s great that you are taking care of yourself!”*
Failure to acknowledge PEM may cause potential iatrogenic harm
*“Became bedridden for 1 year after rehabilitation because I had to exercise four times a day on weekdays. It was not adapted to ME at all. The basic philosophy at the center was that one could become healthy through exercise.”* *“Now, 4 months later, I am still worse than when I went to the rehabilitation institution. But the place is very good; one just has to be healthier than I was to benefit from the stay.”*
New knowledge and strategies may take time before potential benefit is recognized
*“It took a long time, approximately 6 months, before there was an effect of the changes I made.”*

When evidence for curative treatments for ME/CFS is lacking, intervention should at least aim at educating the patient to optimize their ability to maintain function in everyday activities and reduce PEM. This may help to alleviate symptoms and increase quality of life (35, 36). Therefore, in updated clinical recommendations for ME/CFS, educational approaches are included. They typically aim at empowering the patient for self-management with a focus on pacing strategies to conserve energy and focus on coping with a disease with substantial function loss and symptom burden.

In the rehabilitation survey, applied intervention methods were not evaluated systematically, but the programs are usually multidisciplinary and patient education is often part of a rehabilitation program. In the case PEM was addressed in the rehabilitation, nearly three-quarters of respondents reported that they had learned a lot which they had benefited from afterward. This applied to less than a third of the patients if PEM had not been discussed.

In the hospital survey, a considerable portion of the respondents had received educational group courses as well. Some patients received educational courses that were aimed exclusively at ME/CFS patients, while others were included in courses aimed at patients with more general fatigue or health problems. Nearly all participants of ME/CFS-specific courses reported to have obtained information about PEM, but only one of seven participants of the less specific courses reported the same. Apparently, the focus on education, and counseling had been delivered from clinical settings with different explanatory approaches to ME/CFS. Not informing ME/CFS patients about their main disabling symptoms is both worrying and unacceptable and may lead to severe consequences for the patients. In our study, functional deterioration was reported by over seven out of 10 participants of the non-specific courses, but only by less than two out of 10 of the participants of the ME/CFS-specific education. Understandably, the perceived impact on health and functioning and rated care quality was associated with this. Half of the patients who had not received information about PEM during their hospital intervention, versus only one in each 20 patients who had received this, felt that they had been treated incorrectly.

Generally, in intervention effect studies, clinical effectiveness is evaluated. Unfortunately, as reported in our study, even when PEM was addressed in the therapeutic approach, clinical improvements were generally absent. Due to the nature of the disease, some deterioration can be expected after out-of-home interventions, particularly among patients with higher disease degrees. The combined burden of travel, social interaction, coping with time schedules, etc. will often be far beyond the patients’ day-to-day activity level.

Compared to our study, higher improvement rates were reported following specialist ME/CFS services in England (70). At 1-year follow-up, 28% reported overall improvement, and only 8% worsened health. One reason might be that the specialist services are indeed better tailored to this specific patient group. Other reasons might be that the evaluated patients had a shorter duration of ME/CFS and were only mildly affected (70). Our data did not cover treatment at a specialist ME/CFS service.

### 4.2 Is addressing PEM related to the explanatory view of me/CFS?

The PEM phenomenon challenges existing medical assumptions of the health benefits of exercise and other physical and mental activity and sensory stimuli (71). As knowledge and understanding of PEM are crucial for diagnosis and maintaining optimal functioning in ME/CFS, early screening and explaining explicitly about PEM are essential in clinical consultations where ME/CFS is suspected (14, 35). Failure to recognize ME/CFS and PEM may result in poor management in daily life and in the clinical approach, which may hamper recovery potential and aggravate the disease (62, 72).

Only two out of five respondents had noticed that PEM had been addressed in the clinical consultations. The main reason for not discussing PEM in a clinical consultation is probably that the clinician does not acknowledge PEM as an essential feature in ME/CFS. The applied explanatory model in the various clinical settings was not explicitly evaluated in the surveys. However, not acknowledging PEM as a key phenomenon, which in this study was associated with little focus on the patients’ symptoms and poor specific suitability of intervention, is in our opinion an obvious indication of a psychosomatic view. Some of the citations confirm an apparent psychosomatic approach at some of the evaluated healthcare services (see Table 6).

One of the assumptions derived from a biopsychosocial perspective is the sustained arousal hypothesis (73), based on ‘the cognitive activation theory of stress’ (CATS) (74). According to CATS, the sustained stress responses may originate from different precipitating factors (interacting with predisposing factors (genetic traits, personality) and learned expectations (classical and operant conditioning)). Although this theory has not been confirmed, the sustained arousal hypothesis has strong support in Norway, including in some of the evaluated departments, as mirrored in some of the comments (see Table 6).

Because of the presence of a strong psychosomatic network in Norway (3), and the equivocal explanatory view and recommendations for approaching ME/CFS of both the National Advisory Unit on CFS/ME and the Norwegian CFS/ME guidelines (41), it was not surprising to meet a psychosomatic view in several of the specialist healthcare services evaluated in our study.

The respondents’ own underlying assumptions explaining their symptoms had not been assessed. However, for majority of the ME/CFS patients, a predominantly biomedical explanation of their disease usually fits their experiences better than a psychosomatic approach (32, 75, 76). Generally, many ME/CFS patients feel that the doctors psychologize too much, trivialize the symptoms, or tell them that their symptoms are psychosomatic (43, 77–79). If patients meet an opposing explanatory model in healthcare practice, negative patient experiences and dissatisfaction with received care may arise (75, 79). In our study, failure to address PEM led to ineffective, harmful healthcare and respondents reported poor disease understanding of ME/CFS among healthcare providers and a lack of validation of their illness experiences (see also Table 6). This has also been reported in previous studies (42, 43, 45, 79, 80). The high internal consistency of not addressing PEM and a reported approach that was poorly customized to ME/CFS suggests that these elements may measure a similar notion of viewing ME/CFS (58).

Illnesses that lack clear pathophysiology, that has inconsistent diagnostic criteria, inadequate research focus, and lack of proper training, seem frequently to be related to negative consequences or iatrogenesis for the patient (80–82). As in our study, Geraghty and Blease (32) recognized several modalities of iatrogenesis in ME/CFS such as high levels of patient dissatisfaction, challenges to the patients’ narratives and experiences, and negative responses to therapy. In addition, other modalities were identified, such as difficulties in reaching an acceptable diagnosis of ME/CFS and access to medical care and social support.

### 4.3 Methodological issues, strengths, and limitations

To our knowledge, this is the first study to evaluate the significance of addressing PEM in the clinical approach to ME/CFS patients in naturalistic settings of specialist healthcare practice. The evaluation of PEM-focus was in fact not the primary outcome of the initial surveys. This may have reduced respondent bias because they were unaware of the aim of the present analyzes of assessing the significance of acknowledging the PEM phenomenon with regard to their health and perceived care quality.

The inclusion of two comparable surveys, together covering specialist healthcare for ME/CFS patients in Norway, and the large sample size from a large geographical area in the rehabilitation survey were also strengths of this study. Another key feature of this study is the focus on intervention-induced ‘deterioration’ versus ‘no-deterioration’ instead of evaluating clinical effectiveness. This seems especially relevant in the evaluation of ‘real-life’ interventions for ME/CFS because of general limited improvement in health status. Instead, exacerbations are frequently described in patient surveys but usually ignored or camouflaged in the presentation of average scores.

In the analyzes of our study, the occurrences of provided healthcare are in fact the main study focus and not the individual respondents. Therefore, in the hospital survey, some respondents assessed their experiences from more than one department. These have been analyzed as independent occurrences. We considered this as acceptable since ME/CFS is a chronic disease with very limited recovery potential (35, 72, 83), the provided healthcare could cover a time frame of 5 years and the order in which the setting was evaluated was random. Notably, each respondent could evaluate each department only once. The patients’ view concerning PEM-focus and outcome seemed independent of order and number of assessed settings.

This current study has some limitations, mainly concerning methodological issues. The low sample size of the hospital survey may have reduced the statistical power and the chance of detecting true consequences. This might especially concern the analyzes concerning the impact of the interventions on health. In addition, it limited the opportunity to conduct analysis more specific per clinical specialty. Furthermore, the limited diversity of potential covariates in the available data reduced the number of possible factors of interest to adjust for in the regression analyzes.

As a consequence of performing non-prespecified analyzes based on an exploration of two retrospective surveys, some applied measures and scales were not optimal and inconsistent. This applied also to the assessment of PEM-focus that was operationalized with different wording and different scales for the three types of healthcare settings. However, we do not expect this to be a major drawback. Additionally, we were not able to assess the actual focus on PEM in the clinical settings. We were dependent on patients’ perception of its acknowledgment and recall bias may have occurred. This may also have affected the outcome measures that assessed satisfaction and impact on functioning and health status. The retrospective design, however, might have been a methodological plus as the participants gained the opportunity to put their experiences into a longer-term perspective. It may take time to implement new knowledge and learned strategies in daily life before the potential benefit is recognized (see Table 6). Psychosomatic approaches may aim at influencing how patients interpret and report their health state and thus may easily bias subjective outcome measures immediately after the intervention.

A strength of recruiting respondents outside the healthcare settings and collecting anonymous feedback is a better chance of obtaining objective opinions. Patients may hesitate to share negative experiences with healthcare providers because they fear they will appear unmotivated and non-cooperative. This could negatively affect the approval of health benefit allowances.

The recruitment method with open online surveys may, however, have affected the representativeness of the study population. Because of the anonymity, diagnoses could not be verified. ME/CFS status was self-reported by the respondents, therefore is misclassification possible (53). We have limited descriptive data on the respondents, and we have no insight into the population of eligible patients who have visited the hospitals or rehabilitation institutions in the studied period. Invitation of participation to the surveys was shared online among groups that are interested in ME/CFS. However, subjects who are active on social media or are members of the Norwegian ME Association may be overrepresented. Former ME/CFS patients had the possibility to participate in the hospital survey as well. However, none had selected the diagnostic alternative ‘had a fatigue illness before but not now’. In the rehabilitation survey, 20 respondents (0.9%) were excluded because they were neither a patient nor a relative. Some might have been former patients.

Notably, patients with a severe or very severe degree of the disease are poorly represented. An obvious reason is that this group of patients might be less active on social media and has limited energy to answer a questionnaire. They are also less likely to have obtained secondary healthcare because their severe disease status might hamper access to specialist healthcare. In the region of the hospital survey, ambulant healthcare services are not available for this patient group. Challenges in obtaining adequate healthcare have been confirmed in a recent Norwegian study where this was the case for around seven out of ten ME/CFS patients with a severe or very severe sickness degree (84). Some respondents reported that they no longer dared to have contact with healthcare providers due to frequent negative experiences with various healthcare providers.

The hospital survey had aimed at including long COVID patients as well but did not succeed in this. Only two long COVID patients with PEM are part of the study population. Although a relatively high proportion of long COVID patients are expected to develop ME/CFS (47, 85–87), this was not common knowledge at the beginning of 2022, and many long COVID patients with ME/CFS symptoms may not have identified themselves as an ME/CFS patient.

### 4.4 Implications for research and clinical practice

Quality of healthcare is typically described in terms of clinical effectiveness, patient safety, and patient experience. This study evaluated ‘real-life’ experiences of ME/CFS with routine specialist healthcare service in a country with generally high-quality healthcare. The quality of care services delivered to ME/CFS patients seemed strongly related to the acknowledgment of the disease and its cardinal symptom PEM in particular. Ignoring PEM in the approach of ME/CFS appears as a reckless maltreatment of patients.

The findings seem relevant for long COVID as well. Alertness to the possibility of the development of COVID-induced PEM and ME/CFS is, therefore, essential in patients with post-COVID symptoms. In patients with (suspected) ME/CFS or long COVID, early identification and management of PEM may be a cost-effective and the most important method for stabilizing symptoms and improving prognosis and patients’ quality of life (10, 35, 87–89).

In general, ME/CFS-specific knowledge seems limited in many healthcare providers (80, 81, 90–93) and usually ignored in their education (93). The reported iatrogenesis may be traced back to this but also to the fact that at present, ME/CFS is not covered by a defined clinical specialty. As seen from our study, patients had been referred to several medical specialties, both for clinical consultations and intervention. Although ME/CFS is regarded as a multisystem disease, with a neuroimmunological base, often proceeded by an infection, neither the disciplines of infectious diseases, immunology, nor neurology has claimed ‘ownership’ over the diagnosis. This ‘orphaned’ position may have significant implications for whether medical specialists feel an interest or obligation to keep up to date in the field. This might be a reason that still, among many healthcare providers, skepticism is established about whether the disease is primarily ‘physical’ (80, 81, 90, 91). This affects care quality. It has been demonstrated that health providers’ view of ME/CFS being a psychosomatic disorder is associated with worse outcomes than views of ME/CFS as a physical illness (38). Immediate large-scale investment in updated education of (future) healthcare providers about the management of ME/CFS, long COVID, and PEM is essential. In our study, the inter-variability between the departments of how patients rated PEM-focus and related care quality was substantial. This provides opportunities to learn from each other’s clinical practice if interested and open-minded about alternative approaches to ME/CFS.

In healthcare, there is a growing need and recognition of patient experiences as an important aspect of evidence-based practice. Patient experiences as described in our study may contribute to the improvement of the quality of specialist healthcare practice for ME/CFS. The significance of acknowledging the PEM phenomenon for outcome and healthcare quality in ME/CFS or long COVID has not been studied systematically before. It seems unethical to study this in an experimental design, therefore evaluating this in pragmatic settings seems most appropriate. The analyzes and findings presented here can be considered exploratory. Further well-designed research is needed to validate these findings and investigate the value of acknowledging PEM in the approach of ME/CFS and long COVID.

## 5 Conclusion

Despite the inclusion of PEM as a core symptom of ME/CFS in updated diagnostic criteria sets, and the biomedical evidence of the existence of the phenomenon, PEM is still not always accepted and taken into consideration in specialist healthcare practice in Norway.

PEM was not addressed in more than half of the evaluated consultations and rehabilitation stays, and one-third of the hospital interventions. Not addressing PEM doubled the probability of a decline in health and functioning following the intervention and was strongly associated with reduced perceived care quality, satisfaction, and benefit. Acknowledgment of PEM by the healthcare provider was correlated with a more positive rating by the patients of the healthcare providers’ recognition of patient’s symptoms, level of ME/CFS knowledge, and suitability of the intervention to their condition.

This study confirmed the significance of acknowledging the PEM phenomenon in the clinical approach of ME/CFS patients in specialist healthcare practice. When disregarding the PEM phenomenon, healthcare for ME/CFS patients can be described as ineffective, harmful, and of poor quality. In this respect, it seems essential to raise awareness among healthcare providers in specialist healthcare about ME/CFS and PEM.

## Data availability statement

The raw data supporting the conclusions of this article will be made available by the authors, without undue reservation.

## Ethics statement

Ethical approval was not required for the studies involving humans because the study was based on the anonymous replies on two online surveys. The studies were conducted in accordance with the local legislation and institutional requirements. Written informed consent for participation was not required from the participants or the participants’ legal guardians/next of kin in accordance with the national legislation and institutional requirements because the study was based on anonymous replies.

## Author contributions

MW conceptualized and designed the study, conducted the hospital survey and the analyzes related to the hospital survey, and wrote the first draft of the manuscript. SR conducted the analyzes related to the rehabilitation survey and contributed to the final draft of the manuscript. All authors contributed to the article and approved the submitted version.
